# The Smallpox Outbreak

**Published:** 1911-03-25

**Authors:** L. Loat

**Affiliations:** Secretary National Anti-Vaccination League. 27 Southampton Street, Strand, W.C.


### Editor's Letter-Box.

### THE SMALLPOX OUTBREAK.

Sir,—I am not writing to challenge your statements regarding vaccination itself. If I did so you would not admit that a layman was competent to deal with them. But laymen are as competent as medical men to deal with actual facts, and there are two pronouncements in your article on "The Smallpox Outbreak," in your issue of the 11th inst., which are quite untrue. First, your statement that "there are fewer cases of smallpox annually in Germany than there have been already in London alone this past month." Why did you not turn up the Registrar-General's returns before you penned that statement? If you had looked on page 133 of the last Report (that for 1909) you would have found that in the German Empire, from 1892 till 1908, not a single year passed without some deaths from smallpox occurring. In the years 1906, 1907, and 1908 there were 175 such deaths, and if you reckon this represents a fatality rate of 10 per cent. (as pro-vaccinists alleged the vaccinated smallpox fatality was in the last London outbreak) you have 1,750 cases, or nearly 600 per annum. Even if you take only Prussia, we find 132 deaths in the four years ending with 1909, representing 1,300 cases, or 320 cases annually. What are the facts regarding smallpox cases in London alone this past month? Mr. Burns told Mr. Astor on Monday that up to Saturday there had been 54 cases, or about one-sixth of the annual average in Prussia alone, in the four last years for which we have the figures.

Secondly, that "at the end of last week it appeared that of the patients under eight years of age not one had ever been vaccinated." "At the end of last week," signifies up to March 4, and on February 23 Annie Louise Closen and Dorothy Treble, both aged four years, and both vaccinated, were removed from the infirmary suffering from smallpox. On March 2 Norris Welt, aged five years, was removed to the hospital suffering from smallpox, and we have a copy of his vaccination certificate.

If the case for vaccination is as overwhelming as you assume, it cannot suffer if the true facts are given. As a matter of fact it is only overwhelming when the true facts are concealed.—Yours obediently, L. Loat,
Secretary National Anti-Vaccination League.
27 Southampton Street, Strand, W.C.,
March 16.

